# Supplementary material for: Socioeconomic inequalities in utilizing facility delivery in Bangladesh: A decomposition analysis using nationwide 2017–2018 demographic and health survey data
Source: PLoS One. 2022 Nov 28;17(11):e0278093. doi: 10.1371/journal.pone.0278093 (PMC9704681; doi:10.1371/journal.pone.0278093)
Supplement: Attachment — Submitted filename: PONE-D-22-13608_Review.docx [file pone.0278093.s001.docx]

**Reviewer Report**

**Socioeconomic Inequalities in Utilizing Facility Delivery in Bangladesh: A Decomposition Analysis Using Nationwide 2017-2018 Demographic and Health Survey Data.**

The authors have chosen appropriate topic for the study as high maternal mortality is one of the challenge before achieving sustainable development. This can be reduced by pushing maternal healthcare and one among them is institutional delivery/facility based delivery. Authors’ efforts are highly appreciated but there are some suggestions for the improvement of the quality of the paper along with expectation about newness in the paper-

1. The research study aimed to examine socio-economic inequality in facility delivery in Bangladesh using latest available cross sectional data for 2017/18. It’s fine. But complexity of framework is missing somewhere as authors included demographic to socio-economic, regional to empowerment & autonomy to programme factors. So, broadly we can say demand and supply side factors. Even with the demand side factors, authors should follow appropriate framework and including so many dimensions seems not needed.

2. In the inequality measurement section, the authors have elaborated much about the advantages of CI and CC, as it is well known and accepted measure of inequality along income/wealth score. The authors are advices to compress it.

3. In line number 233/234, ‘The CIX accepts values between one and one’- this should be corrected as ‘The CIX accepts values between minus one and plus one’.

4. Why two categories for decision making power and wife beating whereas three category for mother’s enlightenments.

5. Line number 195-239, not needed too much elaboration as well as repetition. Be concise with more citation and studies pertaining to it.

6. In table 2; check for residence, decision making power, mother entitlement, wife beating etc as they are some mistake when analysing distribution of facility based delivery on wealth index category.

7. In table 3, check for wealth status.

8. It table 4, possibly, authors have missed to add the information about the variable on which the model is adjusted for as the cOR and AOR seems having differential magnitude of relative risk. So, it would be really helpful for the readers, if authors can add about the adjustment made into the logistic regression.

9. “While Figure 2 illustrates the inequalities in facility delivery based on one's wealth status. Due to the fact that the concentration curve is above the line of equality, facility delivery was disproportionately higher among women from affluent groups. The relative CIX value for facility delivery is shown in Table 5. A positive estimated CIX in suggests that facility delivery was more concentrated among wealthy women than among poorer women”- The statement made based on the figure 2 is incorrect. The CC appears above the line of equality, when the outcome of interest is high among the poor. In the figure 2, the CC is below the line of equality. Even in table 5, the sign of CI value is positive. So, be careful when interpreting such inequality measure. So, please do correct it.

10. Authors are advised to interpret the results of table 5 [decomposition of CI]as it is missing in the manuscript.

11. Figure 1 needs appropriate formatting, look it carefully.

12. Line 356-360 pertaining to women work status and place of delivery. In case of women work, type of work regulates more to access to services rather working or not working status. Think the women who are working but are engaged in agricultural activities to those who are in white collar jobs. So, the findings on those lines are also needs corroboration.

13. Discussion section mostly revolved around education and economic status. Authors should have a thought on it and restructure it.

14. In the acknowledgement section, Lorenz curve is typed which we draw in case of Gini based inequality but when we use Concentration Index, the curve is concentration curve.

14. Proper editing is also needed.

xxxxxx
