# Supplementary material for: Socioeconomic inequalities in utilizing facility delivery in Bangladesh: A decomposition analysis using nationwide 2017–2018 demographic and health survey data
Source: PLoS One. 2022 Nov 28;17(11):e0278093. doi: 10.1371/journal.pone.0278093 (PMC9704681; doi:10.1371/journal.pone.0278093)
Supplement: Attachment — Submitted filename: Authors Response to Reviewers.docx [file pone.0278093.s002.docx]

**Author’s response to reviewer’s comments.**

PONE-D-22-13608

Socioeconomic Inequalities in Utilizing Facility Delivery in Bangladesh: A Decomposition Analysis Using Nationwide 2017-2018 Demographic and Health Survey Data

We thank the editor and reviewers for their comments and appreciate the care taken in reviewing our manuscript. We hope that our response provided below adequately addresses the concerns of the respective reviewers. We have also taken the opportunity to correct all outstanding grammatical errors and language editing.

|  | **Reviewer’s comments** | **Author’s Responses** |
| --- | --- | --- |
| Reviewer #1 | The authors have chosen appropriate topic for the study as high maternal mortality is one of the challenges before achieving sustainable development. This can be reduced by pushing maternal healthcare and one among them is institutional delivery/facility-based delivery. Authors’ efforts are highly appreciated but there are some suggestions for the improvement of the quality of the paper along with expectation about newness in the paper- | Thanks for your appreciation and acknowledgment. |
|  | 1. The research study aimed to examine socio-economic inequality in facility delivery in Bangladesh using latest available cross-sectional data for 2017/18. It’s fine. But complexity of framework is missing somewhere as authors included demographic to socio-economic, regional to empowerment & autonomy to programme factors. So, broadly we can say demand and supply side factors. Even with the demand side factors, authors should follow appropriate framework and including so many dimensions seem not needed. | Based on the previous literature, we have much discussed about the associated determinants of facility delivery in the introduction section. As we have already provided enough evidence as well as the justification of the inclusiveness of our explanatory variables, another conceptual framework is mandatory. However, we appreciate your comment and we tried to enrich this section in the introduction further. |
|  | 2. In the inequality measurement section, the authors have elaborated much about the advantages of CI and CC, as it is well known and accepted measure of inequality along income/wealth score. The authors are advices to compress it. | Thanks for your comments and suggestions. We elaborate and discuss in details so that researchers in this field can easily understand the details of inequality analysis. However, we consolidated as best as possible by our side. |
|  | 3. In line number 233/234, ‘The CIX accepts values between one and one’- this should be corrected as ‘The CIX accepts values between minus one and plus one’. | Sorry for the unwanted mistake, we have now corrected it. |
|  | 4. Why two categories for decision making power and wife beating whereas three categories for mother’s enlightenments. | We have created it based on a reference paper doi: <https://10.1186/s42506-020-00041-2> |
|  | 5. Line number 195-239, not needed too much elaboration as well as repetition. Be concise with more citation and studies pertaining to it. | Sorry for our repetition. We have checked and deleted the repetition and extra explanation. |
|  | 6. In table 2; check for residence, decision making power, mother entitlement, wife beating etc as they are some mistake when analysing distribution of facility based delivery on wealth index category. | We have checked and didn’t find any anomalies in percentages. |
|  | 7. In table 3, check for wealth status. | We have checked the dataset and no errors we noticed. |
|  | 8. It table 4, possibly, authors have missed to add the information about the variable on which the model is adjusted for as the cOR and AOR seems having differential magnitude of relative risk. So, it would be really helpful for the readers, if authors can add about the adjustment made into the logistic regression. | The details of the statistical modelling adjustment has been described under ‘Statistical analysis” section. Line-179-183 |
|  | 9. “While Figure 2 illustrates the inequalities in facility delivery based on one's wealth status. Due to the fact that the concentration curve is above the line of equality, facility delivery was disproportionately higher among women from affluent groups. The relative CIX value for facility delivery is shown in Table 5. A positive estimated CIX in suggests that facility delivery was more concentrated among wealthy women than among poorer women”- The statement made based on the figure 2 is incorrect. The CC appears above the line of equality, when the outcome of interest is high among the poor. In the figure 2, the CC is below the line of equality. Even in table 5, the sign of CI value is positive. So, be careful when interpreting such inequality measure. So, please do correct it. | Thanks for pointing this out. We are sorry for this unwanted mistake; we have corrected it. |
|  | 10. Authors are advised to interpret the results of table 5 [decomposition of CI]as it is missing in the manuscript. | Line 259-267 presented the description of the Table 5 results of decomposition of CIX. |
|  | 11. Figure 1 needs appropriate formatting, look it carefully. | Done. |
|  | 12. Line 356-360 pertaining to women work status and place of delivery. In case of women work, type of work regulates more to access to services rather working or not working status. Think the women who are working but are engaged in agricultural activities to those who are in white collar jobs. So, the findings on those lines are also needs corroboration. | References added. |
|  | 13. Discussion section mostly revolved around education and economic status. Authors should have a thought on it and restructure it. | Since the prime objective of this study was to measure the socio-economic inequality…therefore we concentrated more in discussing the discussion based on the findings of wealth and education status. |
|  | 14. In the acknowledgement section, Lorenz curve is typed which we draw in case of Gini based inequality but when we use Concentration Index, the curve is concentration curve. | Thanks for noting this. We corrected it. |
|  | 14. Proper editing is also needed. | We have checked the paper and all possible errors have been solved. |
|  |  |  |
| **Reviewer #2** |  |  |
|  | The authors attempt to study an important issue in the context of developing countries. This study comprehensively analyzed the factors determining the use of facility delivery in Bangladesh. However, before its acceptance, this study needed major revision to make it more apparent to the readers. | Thanks for your comments. |
|  | The author provides a strong background for the study; however, it needs to add the following points in this section.  1. The authors should emphasize the relevance of this study in the context of Bangladesh. 2. The evidence of the socioeconomic inequality in the utilization of facility delivery from the previous study is missing in the background. The authors needed to provide an explicit summary of what is already known from the earlier studies and the additional contribution of the current research on this topic. | We have tried to best to recapture the existing inequality and problems associated with place of delivery in the LMICs. In addition, due to safe delivery how many fatalities have been occurring around the world has been described in the Background section. |
|  | Data sources:  In the data section, the author needed to provide some information about the data, such as the number of households, the number of women interviewed, and the sample size. | Line 150-153 described details. |
|  | The outcome variable study period is missing from the manuscript. The reference period of the outcome variable must be mentioned in detail of the outcome variable, whether facility delivery is analyzed for the last birth or the birth in some specific years. | Thanks for pointing this out. It would be “last”. Line 157 |
|  | Explanatory variable:  The justification for using the explanatory variable in the study is missing. Particularly how the mother's age and wife's Beating are associated with the delivery facility's utilization, these variables are also not interpreted in the results and discussion sections. In addition to the controlled variables, the model can control for any complications before the delivery if any information is given in the data. | Thanks for your comments. During literature review we have noticed that previous study found an association with these variables, that’s why we included in our study.  We didn’t extend the discussion because our intention was to explore the socio-economic inequality therefore, we focused to describe the socio-economic variables for discussion. |
|  | In the methods section, a few lines are repeated; for example, lines 211-214 and 221-124 are repeated and also needed to make clearer these sentences. | Sorry for the unwanted mistake. |
|  | In the equations, details of each component should be cleared to the readers; for example, what's mean is μ. | Line 241-243 clearly described the equations. |
|  | Results: The authors used unadjusted logistic regression, but there is no single sentence about the results in the result section. Moreover, the results from the models and decompositions needed to give more interpretation. | Line 189-192, where we mentioned we interpreted only adjusted model results which is very common in public health literature.  In addition, line 286-294 shows the interpretation of the decompositions results. |
